# Predicting the Response of Molluscs to the Impact of Ocean Acidification

**DOI:** 10.3390/biology2020651

**Published:** 2013-04-02

**Authors:** Laura M. Parker, Pauline M. Ross, Wayne A. O’Connor, Hans O. Pörtner, Elliot Scanes, John M. Wright

**Affiliations:** 1 School of Science and Health, University of Western Sydney, Hawkesbury K12, Locked Bag 1797, Penrith, Sydney, New South Wales 2751, Australia; E-Mails: pm.ross@uws.edu.au (P.M.R.); 16745966@student.uws.edu.au (E.S.); j.wright@uws.edu.au (J.M.W.); 2 Industry and Investment NSW, Port Stephens Fisheries Centre, Taylors Beach, New South Wales 2316, Australia; E-Mail: wayne.o'connor@dpi.nsw.gov.au; 3 Alfred Wegener Institute for Polar and Marine Research in the Hermann von Helmholtz Association of National Research Centres e. V. (HGF), Am Handelshafen 12, Bremerhaven, 27570,Germany; E-Mail: Hans.Poertner@awi.de

**Keywords:** mollusc, ocean acidification, elevated CO_2_, calcification, physiology, adults, early-life history

## Abstract

Elevations in atmospheric carbon dioxide (CO_2_) are anticipated to acidify oceans because of fundamental changes in ocean chemistry created by CO_2_ absorption from the atmosphere. Over the next century, these elevated concentrations of atmospheric CO_2_ are expected to result in a reduction of the surface ocean waters from 8.1 to 7.7 units as well as a reduction in carbonate ion (CO_3_^2−^) concentration. The potential impact that this change in ocean chemistry will have on marine and estuarine organisms and ecosystems is a growing concern for scientists worldwide. While species-specific responses to ocean acidification are widespread across a number of marine taxa, molluscs are one animal phylum with many species which are particularly vulnerable across a number of life-history stages. Molluscs make up the second largest animal phylum on earth with 30,000 species and are a major producer of CaCO_3_. Molluscs also provide essential ecosystem services including habitat structure and food for benthic organisms (*i.e.*, mussel and oyster beds), purification of water through filtration and are economically valuable. Even sub lethal impacts on molluscs due to climate changed oceans will have serious consequences for global protein sources and marine ecosystems.

## 1. Introduction

Since the industrial revolution some 200 years ago, fossil fuel combustion, cement production and deforestation have led to a near 40% increase in the concentration of carbon dioxide (CO_2_) in the atmosphere and this will continue to increase over the next century [1,2,3,4]. Approximately one third of all anthropogenic CO_2_ emissions are stored on average, within 100 m of the surface of the oceans [4] with measurable effects on surface ocean chemistry where a slow but continuous equilibrium with the atmosphere exists. To date, absorption of CO_2_ into the surface ocean has led to a global decline in mean pH levels of more than 0.1 units compared with pre-industrial levels [5]. Assuming median emission scenarios predicted by the Intergovernmental Panel on Climate Change [2], a further 0.3–0.4 unit decline is expected over this century as the partial pressure of CO_2_ (*p*CO_2_) reaches 800 ppm [5,6]. At the same time there will be a reduction in the concentration of carbonate ions (CO_3_^2−^) which will lower the calcium carbonate (CaCO_3_) saturation state (Ω) in seawater and mirror the shrinking ocean regions where organisms can deposit CaCO_3_ shells and skeletons [5,7].

A number of recent studies and meta-analyses aimed at determining the impacts of “ocean acidification” on marine and estuarine organisms conclude that the likelihood of severe consequences for calcifying marine and estuarine organisms is high [8,9,10,11,12,13], especially molluscs [14]. Molluscs are major producers of CaCO_3_ in marine and estuarine ecosystems [15,16,17,18] and make up the second largest animal phylum on earth with over 30,000 species [19]. Studies on the responses of molluscs to ocean acidification across a number of life-history stages suggest that larvae and adults will find it more difficult to deposit their calcium carbonate (CaCO_3_) shells and suffer a range of negative impacts including changes in metabolism, acid-base status, reduced reproduction, immune response and survival in a climate-changed more acidic ocean [7,20,21,22] and this will have ecological and economic consequences [14,22]. Such economic consequences need to be viewed from the perspective that the annual global harvest of shellfish is estimated to be worth 10−13 billion USD [7], representing 12–16% of total global seafood consumption [23] and increasing with the global demand for protein [24,25]. Some studies anticipate that, depending on demand and consumption rates in Asia, this value could increase to 100 billion USD [22]. Already in recent decades there have been reports of alarming declines in mollusc populations [12] in North America, linked at least in part to acidification [26]. Since 1978 there has been a 75% decline of adult northern abalone *Haliotis kamtschatkana* (Jonas 1845) populations along the British Columbia coast [27,28]. In the Pacific Northwest coast of the United States, there have also been year-by-year declines in the survival of juvenile “seeds” of the Pacific oyster *Crassostrea gigas* (Thunberg 1793) due to the upwelling of acidified waters [26]. It is predicted that anthropogenic ocean acidification will be the dominant driver of pH and aragonite saturation states in estuaries and coastal regions by the end of this century [29,30,31]. Ecologically, molluscs provide essential ecosystem services including habitat structure for benthic organisms (*i.e.*, mussel and oyster beds), act as a biological filter to purify polluted estuarine water, and are a food source for other organisms [15,32].

## 2. Life Cycle

The impact of ocean acidification on molluscs is predicted to vary depending on the life-history stage and habitat. Like many marine and estuarine organisms, many molluscs are broadcast spawners and have a complex lifecycle which includes fertilisation, embryonic and larval pelagic (early-life history) stages followed by a benthic (sometimes sessile) juvenile and adult stage. Embryos and larval early-life history stages have been shown to have specific environmental needs [33]. Given the different physiological, behavioural and habitat requirements which exist between the larval pelagic and adult benthic stages the effects of ocean acidification on molluscs are likely to differ depending on their stage of development. The first deposition of CaCO_3_ for many mollusc species, begins during early larval development with the deposition of amorphous calcium carbonate (ACC). ACC is a form of CaCO_3_ which lacks crystalline structure and is therefore highly susceptible to dissolution [34]. It is only later that larvae deposit a less soluble form of CaCO_3_. Any effect of ocean acidification at an early-life history stage may have carry-over consequences for individual larvae and larval cohorts [35,36,37,38] which cascade to shape the structure and function of marine ecosystems and adult populations [36,39].

## 3. Hypotheses on Responses of Molluscs

Several hypotheses [13] have been proposed to predict the responses of molluscs to ocean acidification including: (a) molluscs will experience a reduction in calcification with the severity depending on the CaCO_3_ polymorph which they secrete, their capacity to regulate acid-base status and the physicochemical environment of the population; (b) early-life history stages and associated processes such as fertilisation and larval development will be more sensitive than later-life history stages such as juveniles and adults; (c) multi-stressor impacts will exacerbate the effects of ocean acidification; and (d) exposure of adults to elevated CO_2_ during reproductive conditioning will result in positive carry-over effects passed from adults to their offspring and increase the resilience of molluscs to ocean acidification.

In this review, we use a hypothesis-driven approach to predict the impacts of ocean acidification on molluscs, provide a synopsis of the physiological responses of molluscs and identify areas of future research.

(a) Molluscs will experience a reduction in calcification with the severity of this reduction differing depending on the CaCO_3_ polymorph which they secrete, their capacity to regulate acid-base status and the physicochemical environment of the population:

Many molluscs deposit shells made from CaCO_3_ and these shells play a vital role in protection from predators, parasites and unfavourable environmental conditions [40]. One of the first hypotheses to be investigated predicted that ocean acidification would decrease the calcification and growth of mollusc shells and skeletons because ocean acidification reduces the availability of carbonate ions at calcification sites and lowers the aragonite and calcite saturation state (Ω), making it more difficult for molluscs to secrete their shells [3,5,13,41,42]. In this review, 37 out of 41 studies on calcification across various time-scales (hours, days, weeks, months) report significant negative effects on calcification including reduced calcification, growth, strength, increased dissolution of mollusc shells following exposure to elevated CO_2_ [30,40,43,44,45,46,47,48,49,50,51,52,53,54,55,56,57,58,59,60,61,62,63,64,65] (Table 1). Further, proteomic analysis has shown an increase in the expression of proteins associated with the cytoskeleton [66].

One of the first studies to directly examine the effects of ocean acidification on the calcification of bivalve molluscs, Gazeau *et al.* [45], exposed the edible mussel *Mytilus edulis* (Linnaeus 1758) and the Pacific oyster *C. gigas* to *p*CO_2_ concentrations of 740 ppm (pH −0.2 from ambient) for two hours. They found a 25% and 10% reduction in the calcification rate of the mussel and oyster, respectively, at these elevated concentrations which were realistic CO_2_ scenarios predicted by the IPCC over the next century. Reduced shell weight and increased shell dissolution were also reported in the gastropod mollusc *Nucella lamellosa* (Gmelin 1791) following a six day exposure to elevated *p*CO_2_ of 750 and 1,200 ppm (pH −0.2 and −0.45 from ambient) [50]. Similar results have also been found in more chronic exposure experiments [40,43,44,46,48,49,51,52,54]. In a longer term, three month exposure of the bivalve mussel *Mytilus galloprovincialis* (Lamarck 1819) to a pH reduction of 0.75 units there was a slowing of shell growth, possibly caused by dissolution [43]. The authors suggested that this dissolution occurred in an attempt by the mussel to compensate for reductions in extracellular pH caused by an accumulation of CO_2_ in the hemolymph.

Despite the generally negative effects of ocean acidification on the calcification and growth of molluscs, some species exhibit neutral or even positive effects. In a study by Ries *et al.* [48] although calcification decreased at elevated CO_2_ (606–2,856 ppm), in seven out of eight mollusc species including the oyster *Crassostrea virginica* (Gmelin 1791), hard clam *Mercenaria mercenaria* (Linnaeus 1758), soft clam *Mya arenaria* (Linnaeus 1758), whelk *Urosalpinx cinerea* (Say 1822), periwinkle *Littorina littorea* (Linnaeus 1758), conch *Stombus alatus* (Gmenlin 1791) and bay scallop *Argopecten irradians* (Lamarck 1819), there was a positive effect of elevated CO_2_ at elevations of 606 and 903 ppm on the limpet *Crepidula fornicata* (Linnaeus 1758). In the only other limpet species to be studied to date, *Nacella concinna* (Strebel 1908) [54] calcification decreased at elevated CO_2_, albeit that this CO_2_ level was −0.8 pH units from ambient concentrations, nearly double that of Ries *et al.* [48]. In the cephalopod mollusc *Sepia officinalis* (Linnaeus 1758) there was no reduction in growth or calcification of the cuttlebone following a six week exposure to extremely elevated *p*CO_2_ concentrations of 4,000–6,000 ppm (pH −0.9) [67]. In a more recent study, there was a positive response in this species to elevated *p*CO_2_, and increased calcification of the cuttlebone following a six week exposure to elevated *p*CO_2_ of 6,000 ppm (pH −0.9) [68]. Further, the effects of ocean acidification on the calcification and growth of molluscs have been found to differ even across populations of the same species. Studies by Berge *et al.* [60], Gazeau *et al.* [45] and Thomsen and Melzner [51] on the edible mussel *M. edulis* found decreased calcification and/or shell growth in the bivalve mollusc following exposure to elevated *p*CO_2_ (pH −0.2 to −1.0) (44 days, 48 hours and two months of exposure, respectively). In two other studies, however, there was no effect of elevated *p*CO_2_ on the calcification and growth of this species [48,51]. Species-specific responses and variation in responses within species in calcification and growth to elevated CO_2_, raise questions about underlying causes. A number of models have been developed to explain why some molluscs differ in their sensitivity to ocean acidification. These models include the CaCO_3_ polymorph which they secrete [48,69,70], their capacity to regulate acid-base status [71] and the physicochemical environment of the population [51,72].

Sensitivity of molluscs to ocean acidification may depend on the form of CaCO_3_ which they secrete during calcification. Sensitivity has been suggested to increase with CaCO_3_ solubility; shells with high-Mg calcite being most soluble followed by aragonite and low-Mg calcite [13]. A meta-analysis of all marine species (not just molluscs) done by Kroeker *et al.* [13] found that the effects of ocean acidification on calcification varied with different mineral forms of CaCO_3_. Surprisingly, however, they found that organisms with aragonite and low-Mg calcite shells and skeletons were negatively affected whilst those with high-Mg calcite, the most soluble form of CaCO_3_, were not [13]. More recently, Ries [73] assessed whether molluscs preferentially secrete less soluble CaCO_3_ polymorphs into their shell during exposure to elevated CO_2_. Ries [69,73] studied the polymorph mineralogy during exposure of elevated CO_2_ in a range of molluscs, including those which secrete predominately aragonite (hard clam, soft clam, limpet, conch), low-Mg calcite (periwinkle, bay scallop, oyster, blue mussel) and an approximately equal proportion of both aragonite and calcite (bimineralic calcifiers whelk) into their shell. The results showed that only the bimineralic calcifier (whelk *Urosalpinx cinerea*) exhibited changes in polymorph mineralogy, having a greater proportion of calcite in their shell following exposure to elevated CO_2_ [69]. In all other mollusc species tested the CaCO_3_ polymorph secreted remained the same. This study suggested that while some biomineralic calcifiers may exhibit mineralogical plasticity of their shell to provide resilience to elevated CO_2_, it is likely that for most mollusc species, the protective organic matrix surrounding the shell and the ability of the organism to maintain pH of the fluid at the site of calcification may be more important indicators of species resilience to ocean acidification than the CaCO_3_ polymorph which they secrete [69]. Future studies investigating the biomineralogical response of molluscs to ocean acidification should consider the thickness of the organic matrix and the ability of the species to maintain the pH of the fluid at the site of calcification [69].

It has been hypothesised that the capacity of an organism to calcify under elevated CO_2_ may be dependent on the regulation of acid-base status [71]. As stated above cephalopods are able to calcify and even increase calcification during exposure to elevated CO_2_, because of their capacity to ion-regulate and maintain extracellular pH through an increase in extracellular [HCO_3_^−^] [71]. In contrast, bivalve and gastropod molluscs which typically show greater sensitivity than cephalopods to ocean acidification have a decreased capacity to regulate ion and acid-base having a more sessile hypo-metabolic mode of life [41,74,75]. Currently no studies have assessed whether the differences in calcification responses of molluscs depend on differences in the regulation of acid-base status during exposure to elevated CO_2_.

**Table 1:** Summary of the impacts of ocean acidification on juvenile and adult molluscs.

Species	Experiment Duration	CO_2_/pH range	Measured	Impact	CO_2_/pH level that first caused an impact	Author
BIVALVES						
*Acesta excavata* (clam)	4 d	*530*, 33,000 µatm *8.12*, 6.35	Respiration	↓/=	−1.72 (33,000 µatm) −1.72 (33,000 µatm) −1.72 (33,000 µatm)	[161]
			Excretion	=		
			pH_e_	↓		
			pH_i_	↓		
*Argopecten irradians* (scallop)	60 d	*370*, 547, 892, 2,551 ppm *8.15*, 8.02, 7.83, 7.45	Calcification	↓	−0.13 (547 ppm)	[48]
*Chamelea nobilis* (clam)	7 d	n/a	Immune response	Changes	−0.4	[135]
		8.1, 7.7, 7.4				
		+Temp. 22, 28 °C				
		+Salinity 28, 34, 40				
*Chlamys nobilis* (scallop)	2 h	n/a 8.1, 7.7, 7.4	Respiration rate	↓	−0.4	[158]
			Clearance rate	↓	−0.4	
			Excretion rate	↓	−0.4	
*Crassostrea gigas* (oyster)	14 d	*380*, 740 ppmv n/a	Calcification	↓	−0.2 (740 ppmv)	[45]
	30 d	n/a	Shell density	↓	−1.2	[55]
		8.2–5.4	Shell weight	↓	−1.2	
	55 d	*582*, 1480 ppm	pH_e_	↓	−0.39 (1,480 ppm)	[137]
		*8.07*, 7.68	*P*_e_CO_2_	↑	−0.39 (1,480 ppm)	
		+Temp. 15, 25 °C	*P*_e_O_2_	=		
			C_e_CO_2_	↑	−0.39 (1,480 ppm)	
			HCO_3_^−^	↑	−0.39 (1,480 ppm)	
			Ca^2+^	=		
			Na^2+^	↓	−0.39 (1,480 ppm)	
			K^+^	↑	−0.39 (1,480 ppm)	
			Tissue metabolite levels	↓	−0.39 (1,480 ppm)	
			Metabolic rate (+temp.)	↑	−0.39 (1,480 ppm)	
*Crassostrea virginica* (oyster)	15 h	n/a	Calcification (+salinity)	↓	−0.63	[30]
		*8.29*, 7.66, 7.56				
		+Temp 20, 30 °C				
		+Salinity 16, 32				
	14–140 d	*380*, 3500 µatm *8.2*, 7.5	Survival	↓	−0.7 (3,500 µatm)	[40]
			Shell growth	↓	−0.7 (3,500 µatm)	
			Somatic growth	↓	−0.7 (3,500 µatm)	
			Shell ultrastructure	↓	−0.7 (3,500 µatm)	
			Respiration rate	↑	−0.7 (3,500 µatm)	
	60 d	*370*, 547, 892, 2551 ppm	Calcification	↓	−0.13 (547 ppm)	[48]
		*8.15*, 8.02, 7.83, 7.45				
	77 d	400, 700–800 µatm 8.11, 7.97 (Sal 15) +Salinity 15, 30	Survival	↓	−0.14 (700–800 µatm)	[140]
			Tissue energy stores	↓	−0.14 (700–800 µatm)	
			Somatic growth	↓	−0.14 (700–800 µatm)	
			Tissue ATP	=		
			Carbonic anhydrase	=		
	14 d	n/a	Shell dissolution	↑	−0.23	[62]
		*7.9*, 7.67, 7.38, 7.17				
	14 d	*395*, 3,617 ppm	Oxidative stress	↑	−0.8 (3,617 ppm)	[66]
		*8.3*, 7.5	Cytoskeletal proteins	↑		
*Laternula elliptica* (clam)	28 d	*390*, 2,639 µatm	Shell dissolution	↑	−0.6 (2,639 µatm)	[54]
		*8.2*, 7.4				
	120 d	187 ^*^, *438*, 735 µatm 8.32 ^*^, *7.99*, 7.78	Metabolic rate	↑	−0.21 (735 µatm)	[152]
			HSP70 expression	↑	−0.21 (735 µatm)	
			Chitin synthase expression	↑	−0.21 (735 µatm)	
*Mercenaria mercenaria* (clam)	14 d	Ω_arag_ *1.2*, 0.6, 0.4	Survival	↓	−0.6 (Ω_arag_ 0.6)	[153]
		*7.9*, 7.3, 7.0				
	60 d	*370*, 547, 892, 2551 ppm	Calcification	↓	−0.7 (2,551 ppm)	[48]
		*8.15*, 8.02, 7.83, 7.45				
	8 h	*424*, 1,120, 1950 µatm	Calcification	↓	−0.38 (1,120 µatm)	[61]
		*8.02*, 7.64, 7.41				
*Mya arebaria* (clam)	60 d	*370*, 547, 892, 2551 ppm	Calcification	↓	−0.13 (547 ppm)	[48]
		*8.15*, 8.02, 7.83, 7.45				
*Mytilus chilensis* (mussel)	70 d	*380*, 750, 1200 ppm*7.91*, 7.71, 7.57	Respiration rate	↓	−0.34 (1,200 ppm)	[160]
			Excretion rate	=		
			Ingestion rate	=		
			Clearance rate	↓	−0.34 (1,200 ppm)	
			Adsorption rate	↓	−0.34 (1,200 ppm)	
*Mytilus edulis* (mussel)	2 h	*380*, 740 ppmv	Calcification	↓	−0.2 (740 ppmv)	[45]
		n/a				
	30 d	n/a	Shell density	↓	−1.2	[55]
		8.2–5.4	Shell weight	↓	−1.2	
	32 d	*665*, 1,161, 1435, 3316 µatm	Immune response	↓	−0.1 (1,161 µatm)	[144]
		*7.8*, 7.7, 7.5, 6.7	Immune surveillance parameters	=		
	44 d	n/a	Survival	↓	−0.5	[60]
		*8.1*, 7.6, 7.4, 7.1, 6.7	Shell growth	↓	−0.5	
	14–56 d	*385*, 560, 840, 1120, 1400, 4000 µatm *8.05*, 7.89, 7.81, 7.7, 7.56, 7.08	Shell growth	↓	−0.49 (4,000 µatm)	[79]
			Somatic growth	=		
			pH_e_	↓	−0.49 (4,000 µatm)	
			*P*_e_CO_3_^2−^	↓	−0.49 (4,000 µatm)	
			*P*_e_CO_2_	↑	−0.49 (4,000 µatm)	
			HCO_3_^−^	=		
			Ca^2+^	=		
			Na^2+^	↑	−0.49 (4,000 µatm)	
			K^+^	=		
			Mg^2+^	=		
	2 mo	*385*, 1120, 2400, 4000 µatm *8.03*, 7.7, 7.38, 7.14	Shell growth	↓	−0.33 (1,120 µatm)	[51]
			Somatic growth	=		
			Metabolic rate	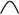	−0.33 (1,120 µatm)	
			NH_4_^+^ excretion rate	↑	−0.33 (1,120 µatm)	
	2 mo	*385*, 1120, 2400, 4000 µatm *8.03*, 7.7, 7.38, 7.16	Shell growth	↑	−0.65 (2,400 µatm)	[59]
			Gene expression (tyrosinases)	↓	−0.65 (2,400 µatm)	
			Gene expression	↓	−0.65 (2,400 µatm)	
			(chitinases)			
	60 d	*370*, 547, 892, 2551 ppm	Calcification	=		[48]
		*8.15*, 8.02, 7.83, 7.45				
	35 d	*472*, 1021, 2114, 3350 µatm	Shell length	=		[65]
		*8.01*, 7.7, 7.4, 7.19	Inorganic shell growth	↓	−0.31 (1,021 µatm)	
		+Food high, low	Organic shell growth	=		
*Mytilus galloprovincialis* (mussel)	7 d	n/a *8.1*, 7.7, 7.4 +Temp. 22, 28 °C +Salinity 28, 34, 40	Immune response	Changes	−0.4	[135]
	78 d	*963*, 1989, 3790 µatm *8.03*, 7.74, 7.48	Somatic growth	↑	−0.3	[159]
			Absorption efficiency	↑	−0.3	
			Ammonium excretion	↑	−0.3	
			Excretion rate	↑	−0.3	
			Clearance rate	=		
			Ingestion rate	=		
			Respiration rate	=		
	3 mo	*0.82*, 3.82 mmHg CO_2_ *8.05*, 7.3	Shell growth	↓	−0.75 (3.82 mmHg)	[43]
			Metabolic rate	↓	−0.75 (3.82 mmHg)	
			pH_e_ level	↓	−0.75 (3.82 mmHg)	
			Protein degradation	↑	−0.75 (3.82 mmHg)	
			HCO_3_^−^	↓	−0.75 (3.82 mmHg)	
			Ca^2+^	↓	−0.75 (3.82 mmHg)	
	84 d	*805*, 1,698, 4,345 µatm *8.13*, 7.84, 7.46	Survival	=	−0.67 (4,345 µatm)	[63]
			Shell growth	↓		
			Tissue growth	=		
*Ostrea edulis* (oyster)	30 d	n/a	Shell density	↓	−1.2	[55]
		8.2–5.4	Shell weight	↓	−1.2	
*Pecten maximus* (scallop)	30 d	*395*, 1,086 µatm	Clapping performance	↓	−0.43 (1,086 µatm)	[165]
		4 °C: *8.19*, 7.76	Aerobic scope	↓	−0.43 (1,086 µatm)	
		10 °: *8.25*, 7.81				
		(+temp. 4, 10 °C)				
*Perna viridis* (green-lipped mussel)	2 h	n/a *8.1*, 7.7, 7.4	Respiration rate	↓	−0.4	[158]
			Clearance rate	↓	−0.4	
			Excretion rate	↓	−0.4	
*Pinctada fucata* (pearl oyster)	2 h	n/a *8.1*, 7.7, 7.4	Respiration rate	↓	−0.4	[158]
			Clearance rate	↓	−0.4	
			Excretion rate	↓	−0.4	
	28 d	n/a *8.1*, 7.8, 7.6	Shell strength	↓	−0.3	[52]
*Ruditapes decussatus* (clam)	75 d	*8.05*, 1698, 4345 µatm *8.13*, 7.84, 7.46	Survival	↑	−0.39 (1,698 µatm)	[72]
			Calcification	=		
			Shell growth	=		
			Weight	=		
	87 d	*730*, 1813, 3702 µatm *8.16*, 7.82, 7.53	Respiration rate	↓	−0.4	[157]
			Clearance rate	↓	−0.4	
			Ingestion rate	↓	−0.4	
			Excretion rate	↑	−0.4	
*Ruditapes philippinarum* (clam)	28 d	n/a	Metal uptake	↑	−1.0	[113]
		*8.5–*6.5				
*Saccostrea glomerata* (oyster)	4 d	*375*, 1000 ppm	Shell growth	↓	−0.36 (1,000 ppm)	[53]
		*8.2*, 7.84				
*Yoldia eightsi* (clam)	35 d	*390*, 2639 µatm	Shell dissolution	↑	−0.8 (2,639 µatm)	[54]
		*8.2*, 7.4				
Community	30 d	*729*–3010 µatm	Structure	↓	−0.3	[138]
		*8.0*, 7.7, 7.3, 6.7				
		(+Temp. 12, 16 °C)				
GASTROPODS						
*Creithium vulgatum* (snail)	ecosystem	*304*–20812 µatm	Shell strength	↓	−0.7	[46]
		*8.17*–6.57				
*Clio pyramidata* (pteropod)	2 d	Ω_arag_ *> 1*, < 1	Shell dissolution	↑	−0.35 (Ω_arag_ >1)	[5]
*Crepidula fornicata* (limpet)	60 d	*370*, 547, 892, 2551 ppm	Calcification	↑	−0.13 (547 ppm)	[48]
		*8.15*, 8.02, 7.83, 7.45				
*Hexaplex trunculus* (snail)	ecosystem	*304*–20812 µatm	Shell strength	↓	−0.7	[46]
		*8.17*–6.57				
*Limacina helicina* (pteropod)	5 d	*350*, 760 µatm	Calcification	↓	−0.3 (760 µatm)	[47]
		*8.1*, 7.8				
	4 - 14 d	4 d: *318*, 940 µatm 4 d: *8.13*, 7.7 14 d: *318*, 883 µatm 14 d: *8.13*, 7.73	Dissolution	↑	−0.43 (940 µatm)	[56]
			Dissolution	↑	−0.4 (883 µatm)	
	29 d	180 ^*^, *380*, 750, 1150 µatm	Survival	↓	−0.43 (1,150 µatm)	[49]
		8.27 ^*^, *8.12*, 7.81, 7.69	Shell growth	↓	−0.31 (750 µatm)	
		(+temp.: 3, 5.5, 8 °C)	Shell degradation	↑	−0.31 (750 µatm)	
*Limacina retroversa* (pteropod)	8 d	280 ^*^, *350*, 750, 1000 ppm	Shell growth	↓	−0.2 (750 ppm)	[57]
		8.2 ^*^, *8.0*, 7.8, 7.6	Shell dissolution	↑	−0.2 (750 ppm)	
*Littorina littorea* (periwinkle)	15 d	n/a *7.97*, 6.63	Predator avoidance	=		[58]
			Predator avoidance (+predator cue)	↑	−1.34	
			Respiration rate	=		
			Respiration rate (+ predator cue)	↓	−1.34	
			Shell thickness	=		
			Shell thickness (+predator cue)	↓	−1.34	
	60 d	*409*, 606, 903, 2856 ppm	Calcification	↓	−0.09 (606 ppm)	[48]
		*8.09*, 8.00, 7.86, 7.42				
	30 d	*428*, 998 µatm *8.03*, 7.67	Shell length	↓	−0.36	[64]
			Shell weight	↑		
			Shell thickness	↓		
			Aspect ratio	↓		
	30 d	*428*, 998 µatm	Metabolic rate	↓	−0.36	[162]
		*8.03*, 7.67	Energy metabolites (ATP)	↓		
*Nacella concinna* (limpet)	14 d	Ω_arag_ *2.66*, 0.47	Shell dissolution	↑	−0.8 (Ω_arag_ 0.47)	[54]
		*8.2*, 7.4				
*Nucella lamellosa* (snail)	6 d	*385*, 785, 1200, 1585 ppm	Shell weight	↓	−0.18 (785 ppm)	[50]
		*7.98*, 7.80, 7.54	Shell dissolution	↑	−0.18 (785 ppm)	
*Osilinus turbinata* (snail)	ecosystem	*304*–20812 µatm	Abundance	↓	−0.7	[46]
		*8.17*–6.57				
*Patella caerulea* (snail)	ecosystem	*304*–20812 µatm	Abundance	↓	−0.7	[46]
		*8.17*–6.57				
*Patella vulgata* (limpet)	5 d	*419*, 2804 µatm *8.2*, 7.6	Metabolic rate	=		[155]
			Protein content	=		
			Feeding rate	=		
			Radula structure	↓	−0.6 (2,804 µatm)	
			pH_e_	=		
			*P*_e_CO_2_	↑	−0.6 (2,804 µatm)	
			HCO_3_^−^	↑	−0.6 (2,804 µatm)	
			Ca^2+^	↑	−0.6 (2,804 µatm)	
			Mg^2+^	=		
*Strombus alatus* (conch)	60 d	*409*, 606, 903, 2856 ppm	Calcification	↓	−0.67 (2,856 ppm)	[48]
		*8.09*, 8.00, 7.86, 7.42				
*Strombus lubuanus* (conch)	6 mo	*360*, 560 ppm *7.94*, 7.90	Survival	↓	−0.04 (560 ppm)	[44]
			Shell growth	↓	−0.04 (560 ppm)	
			Shell weight	↓	−0.04 (560 ppm)	
*Urosalpinx cinerea* (whelk)	60 d	*409*, 606, 903, 2856 ppm	Calcification	↓	−0.09 (606 ppm)	[48]
		*8.09*, 8.00, 7.86, 7.42				
Range of gastropods		*336*–5148 ppm	Settlement	↓	−0.4	[126]
		*8.09*–7.08				
CEPHALOPODS						
*Dosidicus gigas* (squid)	8–12 h	*300*, 1000 ppm				[136]
		*7.93,* 7.62	Metabolic rate	↓	−0.31 (1,000 ppm)	
		+Temp. 10, 20, 25 °C	Activity level	↓	−0.31 (1,000 ppm)	
		+ *P*_O2_ 21, 1%				
*Sepia officinalis* (cuttlefish)	48 h	*493*, 5922 ppm *8.12*, 7.10	pH_e_	↓	−1.02 (5,922 ppm)	[71]
			pH_i_	=		
			*P*_e_O_2_	=		
			*P*_e_CO_2_	↑	−1.02 (5,922 ppm)	
			HCO_3_^−^	↑	−1.02 (5,922 ppm)	
	42 d	*628*, 4271, 6068 ppm *8.01*, 7.23, 7.10	Calcification	=		[67]
			Growth	=		
			Metabolic rate	=		
	42 d	*632*, 6070 ppm	Calcification	↑	−1.0 (6,070 ppm)	[68]
		*8.10*, 7.10				

Bivalves: 37 studies, 20 species (+1 community); Gastropods: 15 studies, 15 species (+1 community); Cephalopods: 4 studies, 2 species. h = hours, d = days, mo = months; control CO_2_/pH listed in italics; ^*^ denotes preindustrial CO_2_ level; 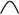 denotes parabolic response.

Finally, the physiochemical environment experienced by molluscs may be pivotal in determining their sensitivity to ocean acidification [6,76,77,78,79]. At Kiel Fjord in the Western Baltic Sea, natural upwelling of CO_2_ rich waters results in an increase in the CO_2_ of the surface ocean for large periods of the year (>2,300 µatm) [79]. Thomsen *et al.* [79] showed that a population of the blue mussel *M. edulis*, a species previously shown in laboratory studies to experience significant reductions in calcification following exposure to elevated CO_2_ [45,51,60], was able to calcify and survive in the acidified conditions. While, the physiochemical environment may indeed be significant in the resilience of molluscs to ocean acidification, the rate of further increases in CO_2_ may exceed tolerance thresholds of molluscs in coastal, estuarine and intertidal environments.

(b) Early-life history stages are more sensitive than juveniles and adults:

The early-life history stages of molluscs, including gametes, embryos and larvae are known to be vulnerable to environmental stressors [80,81] with the number of larvae successfully settling and recruiting into the adult population being extremely low (<10%) [82]. It has been hypothesised that ocean acidification will significantly impact on the early life history stages of molluscs because early-life history stages lack the specialised ion-regulatory epithelia required for maintenance of acid-base status [83] and also deposit CaCO_3_ shells which are comprised of soluble polymorphs such as ACC and aragonite [14,42,84]. The consequences for this on the population is the creation of a bottleneck where genetic variation of the population becomes reduced thereby limiting evolutionary scope for response to ocean acidification [10,14,83,85,86].

Although early studies found negative impacts of ocean acidification on mollusc gametes, embryos and larvae, pH levels were often lower than predicted for marine environments at the end of the century [1,87,88,89,90,91,92]. More recent studies using realistic CO_2_ scenarios have also found similarly negative impacts on fertilisation, embryonic and larval development and settlement in a variety of mollusc species (Table 2, Table 3).

Fertilisation was one of the first processes to be investigated as it was originally predicted to be the most vulnerable stage susceptible to CO_2_-driven ocean acidification [36,93]. For some species, such as the mussels *Mytilus galloprovincialis* [36], *M. edulis* [94] and the subtidal abalone *Haliotis coccoradiata* (Reeve 1846) [95] there was no reduction in fertilisation success during exposure to elevated *p*CO_2_ (*p*CO_2_ of 200 ppm, pH −0.8; *p*CO_2_ of 1,388 to 1,493 µatm, pH −0.5, *p*CO_2_ of 801 to 1,695 µatm pH −0.3 to −0.6 from ambient respectively). In the clam *Macoma balthica* (Leach 1819) and the abalone *Haliotis discus hannai* (Reeve 1846), ocean acidification caused a significant reduction in fertilisation success, but only at CO_2_ levels which have been predicted for the end of this century (pH > −0.4 units) [96,97]. Other studies have reported contrasting results even within the same species, albeit at different geographic locations. For example, there was no reduction in the percentage of fertilisation of the Pacific oyster *C. gigas* in Japan [76] nor in fertilisation, sperm swimming speed and motility in Sweden (*p*CO_2_ 1,000–2,268 ppm, pH −0.34 to −0.8) [77], but there was a reduction in percentage fertilisation of *C. gigas* in an Australian population [78] (Table 2). It is likely that differences in the geographic origin of the molluscs and the design and variation in the methodology in the experiments (sperm concentration, polyandry *versus* single crosses, egg size, gamete quality and sperm-egg contact time) explain some of the variation in the impact of ocean acidification on fertilisation in *C. gigas* [42,76,77,98]. Excessively high sperm concentrations may mask the negative (or positive) impacts of ocean acidification, with low sperm concentration suggested to be a limiting factor for fertilisation during exposure to elevated CO_2_ [14,99]. Pooling of gametes from multiple males and females (polyandry) *versus* single pair male female crosses may also lead to different results [14,100]. Irrespective of the reason, although fertilisation in molluscs appears relatively robust to ocean acidification, our evidence is based on only ten studies on seven species (Table 2). We need more studies on a greater diversity of species before a solid prediction can be made on whether fertilisation in molluscs will be impacted by ocean acidification.

In contrast to fertilisation, early-life history stages have been found to be highly sensitive to ocean acidification (Table 3). Impacts of ocean acidification on the embryos and larvae of molluscs include; smaller sized embryos and larvae [37,38,76,94,97,100,101,102,103,104,105,106,107,108,109,110,111,112], decreased shell thickness [104,107], increased larval development time [36,37,73,78,103,106] reduced survival [37,76,78,96,100,102,111], reduced metamorphosis [108,109], shell abnormalities [12,36,37,76,78,97,101,102,104,105], altered behaviour [106], and alterations in the accumulation of heavy metals [113,114,115] (Table 3).

When the mussel *M. edulis* was exposed to elevated CO_2_ for two days there was a 12.7 ± 0.9% reduction in the shell size of D-veliger larvae and a 24 ± 4% reduction in the hatching success of embryos compared to the controls [102]. Similarly, when the hard clam *Mercenaria mercenaria* and the bay scallop *Argopecten irradians,* were exposed to elevated CO_2_ for 36 days and 52 days respectively, shell thickness decreased by 43%, hinge abnormalities occurred, development time increased and there was decreased survival of larvae in both species [104]. Talmage and Gobler [104] also exposed larvae of *M. mercenaria* and the bay scallop *A. irradians* to a preindustrial CO_2_ level of 250 ppm. Interestingly, larvae reared at this level had greater growth and survival and greater percentage metamorphosis, than larvae reared at both the present day CO_2_ level (390 ppm) and at the elevated CO_2_ (1,500 ppm) level, indicating that either ocean acidification is already impacting larvae of mollusc species [104] or adaptation to present conditions has occurred. In a recent study by Gazeau *et al.* [116], there was no effect of CO_2_-induced changes in pH on the developmental success or growth rates of larvae of the oyster *C. gigas*, as long as carbonate ion concentrations remained above aragonite saturation levels.

Larval molluscs which are smaller with thinner, weaker shells may require a longer length of time in the plankton to have sufficient energy for metamorphosis. A longer larval life may also decrease survival and increase mortality because of increased risk of predation and exposure to other environmental stressors. Talmage and Gobler [104] found a significant decrease in the lipid index of larvae of *M. mercenaria* and *A. irradians* following a 36 day exposure to elevated CO_2_. The authors suggested that the compromised structure of their hinge decreased the ability of larvae to obtain and process food and develop lipid reserves causing alterations in the energy budget to occur at the expense of growth [104].

Reduced survival has been a commonly reported impact of ocean acidification for embryos and larvae of bivalve molluscs following exposure to elevated CO_2_ (Table 3). In a meta-analysis by Kroeker *et al.* [13] the effect of ocean acidification survival was greater in larvae than adults. Reduced survival of larval bivalves including oysters, scallops and clams has been recorded over varying lengths of exposure to elevated CO_2_ (48 h–52 d) [37,38,76,78,102,103,104], although there was no effect on survival of larval *M. edulis* following 13 days of exposure, a reduction of 0.5 units in pH [102].

**Table 2:** Summary of the impacts of ocean acidification on the fertilisation of molluscs.

Species	Experiment Duration	CO_2_/pH range	Measured	Impact	CO_2_/pH level that first caused an impact	Author
BIVALVES						
*Crassostrea gigas* (oyster)	1 h	*432*, 1051 µatm *8.15*, 7.8	Fertilisation	=		[77]
			Sperm swimming speed	=		
			Sperm motility	=		
	2 h	*348*, 2268 µatm	Fertilisation	=		[74]
		*8.21*, 7.42				
	2 h	375, 600, 750, 1000 µatm	Fertilisation	↓	−0.18 (600 µatm)	[78]
		*8.19*, 8.01, 7.93, 7.82				
		(+Temp. 18, 22, 26, 30 °C)				
*Macoma balthica* (clam)	24 h	n/a	Fertilisation	↓	−0.6	[96]
		*8.1*, 7.8, 7.5				
*Mytilus edulis* (mussel)	2 h	*368–381*, 1291–1332 µatm	Fertilisation	=		[94]
		*8.13*, 7.63				
*Mytilus galloprovincialis* (mussel)	2 h	*380*, 2000 ppm	Fertilisation	=		[35]
		*8.13*, 7.42				
*Saccostrea glomerata* (oyster)	2 h	*375*, 600, 750, 1000 ppm	Fertilisation	↓	−0.17 (600 ppm)	[37]
		(+Temp. 18, 22, 26, 30 °C)				
	2 h	375, 600, 750, 1000 µatm	Fertilisation	↓	−0.18 (600 µatm)	[78]
		*8.19*, 8.01, 7.93, 7.82				
		(+Temp. 18, 22, 26, 30 °C)				
GASTROPODS						
*Haliotis coccoradiata* (abalone)	15 min	*327–335*, 814–851, 1051–1104, 1729–1828 µatm	Fertilisation	=		[95]
		*8.25*, 7.9, 7.8, 7.6				
		(+Temp. +2, +4 °C)				
*Haliotis discus hannai* (abalone)	15 h	*450*, 500, 1100, 1650, 2150 µatm	Fertilisation	↓	−0.48 (1,650 ppm)	[97]
		*8.02*, 7.94, 7.68, 7.54, 7.43				

Bivalves: 8 studies, 5 species; Gastropods: 2 studies, 2 species; Cephalopods: 0 studies. min = minutes, h = hours; control CO_2_/pH listed in italics.

**Table 3:** Summary of the impacts of ocean acidification on embryonic and larval development of molluscs.

Species	Experiment Duration	CO_2_/pH range	Measured	Impact	CO_2_/pH level that first caused an impact	Author
BIVALVES						
*Argopecten irradians* (scallop)	19 d	*355*, 681, 1609 ppm *8.08*, 7.83, 7.48	Survival	↓	−0.25 (681 ppm)	[103]
			Development time	↓	−0.25 (681 ppm)	
			Size	↓	−0.25 (681 ppm)	
	52 d	244 ^*^, 387, 739, 1,529 ppm 8.17, *8.04*, 7.80, 7.53	Survival	↓	−0.24	[104]
			Shell thickness	↓	−0.24	
			Shell diameter	↓	−0.24	
			Lipid index	↓	−0.24	
	18 d	240 ^*^, *387*, 773 ppm 8.21, 8.08, 7.81	Survival	↓	−0.27	[108]
			Shell diameter	↓	−0.27	
			Metamorphosis	↓	−0.27	
			Lipid index	↓	−0.27	
	20 d	240 ^*^, *390*, 850 ppm	Survival	↓	−0.1	[109]
			Shell diameter	↓	−0.1	
			Metamorphosis	↓	−0.1	
			Lipid index	↓	−0.1	
*Crassostrea angulata* (oyster)	5 d	*469*, 964, 1447, 2390 µatm *8.08*, 7.81, 7.64, 7.43 (+Salinity 24, 34 ppt)	Shell growth	↓	−0.65	[110]
*Crassostrea ariakensis* (oyster)	30 d	291 ^*^, *386*, 581, 823 µatm	Shell growth	=		[100]
		8.17 ^*^, *8.08*, 7.92, 7.79	Shell calcification	=		
*Crassostrea gigas* (oyster)	48 h	*348*, 2268 µatm *8.21*, 7.42	Survival	↓	−0.79 (2,268 µatm)	[76]
			Size	↓	−0.79 (2,268 µatm)	
			Development rate	↓	−0.79 (2,268 µatm)	
			Shell normality	↓	−0.79 (2,268 µatm)	
	48 h–4 d	375, 600, 750, 1000 µatm *8.19*, 8.01, 7.93, 7.82 (+Temp. 18, 22, 26, 30 °C)	Survival	↓	−0.18 (600 µatm)	[78]
			Size	↓	−0.18 (600 µatm)	
			Shell normality	↓	−0.18 (600 µatm)	
			% Umbonate larvae	↓	−0.18 (600 µatm)	
			% Eyed larvae	↓	−0.18 (600 µatm)	
	24–48 h	n/a 8.2–7.6 (*in situ*)	Early size (24 h)	=	−0.4	[26]
			Mid size (48 h)	↓		
			Shell normality	=		
	6 d	*Control*, 2275 µatm *8.0*, 7.5	Protein expression	↓	−0.5 (2,275 µatm)	[124]
	3 d	*449*, 1020, 2171 µatm *8.03*, 7.72, 7.41	Hatching	=		[116]
			Size	=		
			Calcification	=		
	1–3d	Day 1 *466*, 812, 1150 µatm *7.97*, 7.75, 7.61	Survival	=		[111]
			Size	=		
			Development rate	=		
			Calcification	↑	−0.23 (781 µatm)	
		Day 3 *428*, 781, 1031 µatm *8.00*, 7.77, 7.66	Survival	↓	−0.23 (781 µatm)	
			Size	↓	−0.34 (1,031 µatm)	
			Development rate	=		
			Calcification	↓	−0.34 (1,031 µatm)	
*Crassostrea virginica* (oyster)	30 d	291 ^*^, *386*, 581, 823 µatm	Size	↓	−0.16 (581 µatm)	[100]
		8.17 ^*^, *8.08*, 7.92, 7.79	Calcification	↓	−0.16 (581 µatm)	
	20 d	*355*, 681, 1609 ppm *8.08*, 7.83, 7.48	Survival	↓	−0.6 (1,609 ppm)	[103]
			Size	↓	−0.25 (681 ppm)	
			Development rate	↓	−0.25 (681 ppm)	
	20 d	240 ^*^, *390*, 850 ppm	Survival	↓	−0.2 (850 ppm)	[109]
			Shell diameter	↓	−0.2 (850 ppm)	
			Metamorphosis	↓	−0.2 (850 ppm)	
			Lipid index	↓	−0.2 (850 ppm)	
*Macoma balthica* (clam)	19 d	n/a *8.1*, 7.8, 7.5	Hatching	↓	−0.3	[96]
			Survival	↓	−0.6	
			Size	↓	−0.3	
			Metamorphosis	=		
*Mytilus californianus* (mussel)	5–8 d	*380*, 540, 970 ppm *8.07*, 7.95, 7.75	Shell area	↓	−0.12 (540 ppm)	[107]
			Shell thickness	↓	−0.12 (540 ppm)	
			Shell strength	↓	−0.12 (540 ppm)	
			Tissue mass	↓	−0.12 (540 ppm)	
*Mytilus edulis* (mussel)	13 d	*460*, 1100, 1900 µatm	Survival	=		[102]
		*8.1*, 7.8, 7.6	Hatching	↓	−0.5 (1,900 µatm)	
			Size	↓	−0.3 (1,100 µatm)	
	64 d	*368*, 1291 µatm *8.13*, 7.63	Size	↓	−0.5 (1,291 µatm)	[94]
			Development rate	=		
			Shell normality	=		
			Feeding rate	=		
*Mytilus galloprovincialis* (mussel)	6 d	*380*, 2000 ppm *8.13*, 7.42	Size	↓	−0.71 (2,000 ppm)	[36]
			Development rate	↓	−0.71 (2,000 ppm)	
			Shell normality	↓	−0.71 (2,000 ppm)	
*Mytilus trossolus* (mussel)	60 h	*400*, 1000 ppm *8.3*, 7.9	Shell length	↓	−0.4 (1,000 ppm)	[112]
*Mercenaria mercenaria* (clam)	18 d	*355*, 681, 1609 ppm *8.08*, 7.83, 7.48	Survival	↓	−0.25 (681 ppm)	[103]
			Size	↓	−0.25 (681 ppm)	
			Development rate	↓	−0.25 (681 ppm)	
	36 d	244 ^*^, *387*, 739, 1529 ppm 8.17, *8.04*, 7.80, 7.53	Survival	↓	−0.24	[104]
			Shell thickness	↓	−0.24	
			Shell diameter	↓	−0.24	
			Lipid index	↓	−0.24	
	18 d	240 ^*^, *387*, 773 ppm 8.21, 8.08, 7.81	Survival	↓	−0.27	[108]
			Shell diameter	↓	−0.27	
			Metamorphosis	↓	−0.27	
			Lipid index	↓	−0.27	
*Saccostrea glomerata* (oyster)	48 h	*375*, 600, 750, 1000 ppm	Survival	↓	−0.17	[37]
		*8.20*, 8.03, 7.95, 7.84	Size	↓	−0.17	
		Temp.: 18, 22, 26, 30 °C	Shell normality	↓	−0.17	
	48 h–4 d	*375*, 600, 750, 1000 µatm *8.19*, 8.01, 7.93, 7.82 Temp.: 18, 22, 26, 30 °C	Survival	↓	−0.18	[78]
			Size	↓	−0.18	
			Shell normality	↓	−0.18	
			% Umbonate larvae	↓	−0.18	
			% Eyed larvae	↓	−0.18	
	8 d	*220*, 509, 756 µatm *8.11*, 7.81, 7.64	Survival	↓	−0.3	[101]
			Size	↓	−0.3	
			Shell normality	↓	−0.3	
Range of bivalves		*336*–5148 µatm	Settlement	↓	−0.4	[126]
		*8.15*–7.08				
GASTROPODS						
*Cavolina inflexia* (pteropod)	13 d	*380*, 857, 1713 µatm	Size	↓	−0.28 (857 µatm)	[105]
		*8.1*, 7.82, 7.58	Shell normality	↓	−0.28 (857 µatm)	
*Haliotis cocoradiata* (abalone)	21 h	*306*, 1,077, 1770 ppm	Calcification	↓	−0.4 (1,077 ppm)	[12]
		*8.2*, 7.8, 7.6	Shell normality	↓	−0.4 (1,077 ppm)	
*Haliotis discus hannai* (abalone)	90 h	*450*, 500, 1100, 1650, 2150 µatm *8.02*, 7.94, 7.68, 7.54, 7.43	Hatching rate	↓	−0.34 (1,100 ppm)	[97]
			Survival	=		
			Shell normality	↓	−0.34 (1,100 ppm)	
*Haliotis rufescens* (abalone)	6 d	*380*, 570, 990 ppm	Thermal tolerance	↓	−0.18 (990 ppm)	[123]
		*8.05*, 7.97, 7.87	*Ap24* expression	=		
*Littoria obtusata* (periwinkle)	24 d	*Control*, 1093 ppm *8.1*, 7.6	Viability	↓	−0.5 (1,093 ppm)	[106]
			Development rate	↓	−0.5 (1,093 ppm)	
			Heart rate	↓	−0.5 (1,093 ppm)	
			Altered behaviour		−0.5 (1,093 ppm)	
			Altered shell		−0.5 (1,093 ppm)	

Bivalves: 21 studies, 12 species; Gastropods: 5 studies, 5 species; Cephalopods: 0 studies. h = hours, d = days, mo = months; control CO_2_/pH listed in italics; ^*^ denotes preindustrial CO_2_ level.

**Figure 1:**
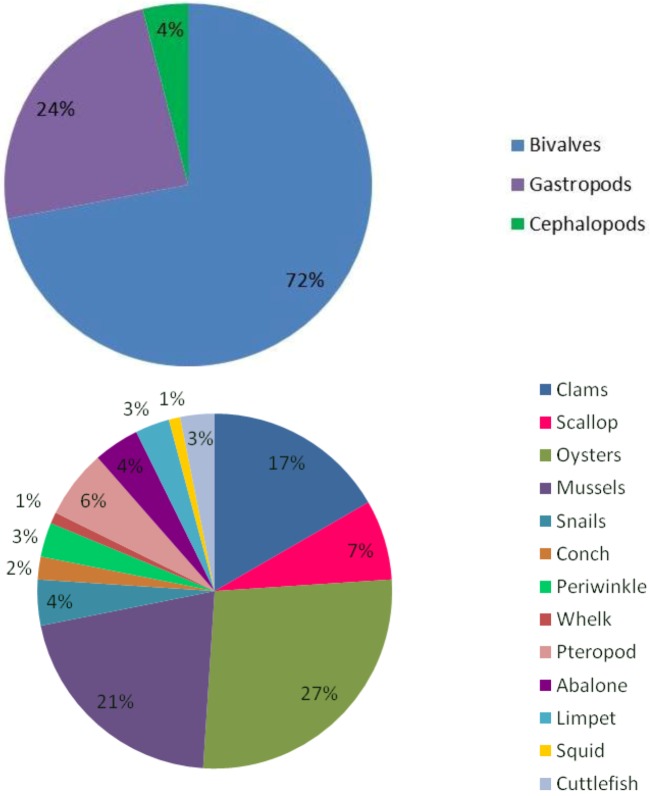
Proportion of studies (as a percentage) done on each mollusc group.

In comparison to bivalves, the effects of ocean acidification on gastropod and cephalopod embryos and larvae have been considerably less studied (Figure 1). In the cephalopod *Sepia offincialis*, unlike juvenile and adult stages which show neutral or positive effects of ocean acidification, eggs are negatively affected, with an increase in the accumulation of heavy metals following exposure to elevated CO_2_ [115]. In one of only two studies on the impact of elevated CO_2_ on the survival of a larval gastropod, Byrne *et al.* [12] found the survival of larval abalone Haliotis coccoradiata decreased while the number of uncalcified larvae and the number of abnormal juveniles (following metamorphosis) increased. In the gastropod snail *Littorina obtusata* (Linnaeus 1758) the viability of embryos decreased following exposure to elevated CO_2_, equivalent to a reduction in pH of 0.5 units. This was accompanied by an increase in development time, reduced heart rate, altered shell morphology, and embryo behaviour [104].

Any variation in the behaviour of larval molluscs during exposure to elevated CO_2_ has largely been ignored to date. This is surprising given the important role that behaviour plays in the survival of a species [42]. Spinning activity in mollusc embryos for example, mixes cellular fluids and increases environmental oxygen delivery to embryos [117]. Rotational behaviour of embryos is also linked to neural development [117,118] which is an essential survival mechanism to detect and avoid predators. Reductions in spinning rate, infrequent activity and less time crawling occurred when embryos of the snail *L. obtusata* were exposed to elevated CO_2_ [106]. Adult gastropods *L. littorea* exposed to elevated CO_2_ were unable to increase the thickness of their shell, a defense mechanism usually employed in the presence of a predator under ambient CO_2_ conditions. The snails did, however, increase their avoidance behaviour at elevated CO_2_, which may partly compensate for their lack of morphological defense [58].

The underlying mechanisms explaining larval responses to ocean acidification have been largely unexplored. For species such as the oyster *C. gigas* [76] and the mussel *Mytilus galloprovincialis* [36], the first effects of elevated *p*CO_2_ on the bivalve species were observed during the trochophore stage which corresponds with the onset of shell mineralization [76,119,120]. Calcification may be a major process affected by elevated CO_2_ [36,76], although other processes such as decreased protein synthesis and metabolism may result in smaller larvae, decreased survival and increased larval development. In other phyla, analysis of gene expression has revealed that calcification (biomineralization) energy metabolism, as well as, thermal and cellular stress responses of larvae were suppressed in the sea urchin, *Strongylocentrotus sp.*, during exposure to elevated CO_2_ [121,122]. Yet in mollusc larvae Zippay and Hofmann [123] found no effect of elevated CO_2_ on the expression of two key shell formation genes in mollusc larvae of the red abalone, *Haliotis rufescens* (Swainson 1822), but they did find the thermal tolerance of some larval stages (pretorsion and late veliger) was reduced as was larval survival. A recent study by Dineshram *et al.* [124] found a reduction in protein expression in larvae of the oyster *C. gigas*. The expression of genes involved in energy and protein metabolism (F-ATPase, hexokinase and elongation factor alpha) and the shell protection/periostracum formation (tyrosinase) increased in adult *M. edulis* exposed to elevated CO_2_ while there was a decrease in chitinase which is important in shell calcification in tissue from the outer and inner mantle [59].

Although the sensitivity of embryos and larvae of molluscs to ocean acidification differs between life history stages, there does not seem to be a hierarchical order of decreasing sensitivity with increasing age as previously thought [14]. For example the thermal tolerance of the abalone *Haliotis rufescen*, was impaired at reduced pH (pH −0.2 from ambient) in the earlier pre-torsion and late veliger life-history stages, but not in post-torsion and pre-metamorphic veligers [123].

Embryos and larvae of molluscs vary in their sensitivity to ocean acidification even between closely related species using identical methodologies and experimental design. In oysters there was a 20% reduction in the survival of larvae of *C. gigas* after 48 h exposure to elevated CO_2_ (pH −0.4 from ambient) compared to a 35% reduction in the survival of larvae of *Saccostrea glomerata* (Gould 1850) [78]. Further, in the oyster *C. virginica* there was a significant decrease in shell growth and calcification after 30 days exposure to elevated CO_2_ (pH −0.15–0.3 from ambient), but there was no effect on the shell growth and calcification of the oyster *C. ariakensis* (Fujita 1913) [100]. Species-specific responses may occur due to differences in the physical and chemical characteristics and dynamics of the environment in which species live and/or the evolutionary history of a species, and require further investigation [100].

Earlier studies used mineral acid to manipulate the carbonate chemistry of seawater while more recent studies use direct addition of CO_2_ into the experimental seawater. Some studies are acute in nature, lasting only hours or days while others are more chronic, lasting weeks or months. Some studies measure the effects of ocean acidification on single embryonic and larval stages, while others measure the cumulative affects across two or more stages (Table 3). Despite differences in methodology in nearly all studies on embryos and larvae to date there have been similar negative results.

Even when embryos and larvae of bivalves and gastropods survive exposure to elevated CO_2_ they must still undergo settlement before they can successfully recruit into the population. Settlement involving the selection of a suitable substratum and a set of morphogenetic changes (metamorphosis) is an energetically costly time which often results in large-scale mortality [125]. If larvae reach the settlement stage with lower energy reserves then both pre- and post-settlement may be affected leading to higher post-settlement mortality and less recruitment into the adult population. Despite the importance of settlement only three studies have measured the impacts of ocean acidification on this vital life-history stage [27,94,126]. In two laboratory studies there was no effect of elevated CO_2_ on the settlement of the mussel *M. edulis* [94] or the abalone *Haliotis kamitschatkana* [27]. In the only field study to date, however, settlement on artificial collectors was reduced in a range of bivalve and gastropod species along a pH gradient of 8.15–7.08 [126].

(c) Multiple stressors will exacerbate the effects of ocean acidification:

Although CO_2_-driven ocean acidification will potentially pose serious consequences for the success and survival of many mollusc species, particularly bivalves and gastropods, it will not act in isolation to other environmental effects [127]. It is predicted, that increased oceanic CO_2_ concentrations over the next century will be accompanied by increased temperature (2–6 °C) [128]. Ocean acidification may act in synergy with other suboptimal environmental conditions including fluctuating salinity (*i.e.*, estuarine habitats) and reduced oxygen levels (hypoxia) [129].

It is hypothesised that the effects of ocean acidification on various invertebrates will be exacerbated by elevated temperature [78,130,131,132,133,134,135]. In the few mollusc species studied to date, most of the evidence suggests that the synergistic effect of elevated CO_2_ and temperature will be greater than the effect of elevated CO_2_ alone [37,49,78,135,136,137,138,139]. Parker *et al.* [37,78], found the size and number of larvae decreased and the number of abnormal larvae increased in the Pacific oyster *C. gigas* and the Sydney rock oyster *S. glomerata* with increasing *p*CO_2_.These deleterious effects were greater at elevated temperature (+4 °C) [37,78]. In *S. glomerata*, exposure to both elevated CO_2_ (pH −0.4 from ambient) and elevated temperature (+4 °C) led to 100% mortality of the larvae after only 24 h. In a mesocosm experiment, Hale *et al.* [138] found that there was a reduction in species diversity and abundance at elevated *p*CO_2_ (pH −0.7 and −1.3 from ambient) which was greater with elevated temperature (+4 °C). Exposure of the jumbo squid *Dosidicus gigas* (d’Orbigny 1835) to elevated *p*CO_2_ caused a significant reduction in SMR at ambient temperature (10 °C). When temperature was moderately elevated (20 °C), SMR of the squid was increased. The upper thermotolerance limit of the squid was reached when the temperature exceeded 25 °C, resulting in metabolic depression [136]. It is likely that the effects of elevated temperature in synergy with ocean acidification are highly dependent on the thermal window [75]. If the thermal window is exceeded, elevated temperature may exacerbate the negative impacts of ocean acidification. If the thermal window is not exceeded, however, elevated temperature may act to suppress or even ameliorate the impacts of ocean acidification. Higher temperature mitigated the impacts of reduced pH (pH −0.5 from ambient) on the calcification of juveniles of the oyster *C. virginica* [30]. Neither ocean acidification nor temperature had an effect on the fertilisation of the abalone *Haliotis coccoradiata* [139]. Elevated temperature also had no additional effect on the altered metal uptake in eggs of the cuttlefish *S. officinalis* that was induced by elevated *p*CO_2_ [115]. In one of the only studies to consider the synergistic effects of ocean acidification and fluctuating salinity, Dickinson *et al.* [140] found weakening of the shells and increased energy consumption in juveniles of the eastern oyster *C. virginica*. While the results of these studies suggest that for many mollusc species, there may be even greater impacts when ocean acidification acts in synergy with other factors such as temperature or salinity than either factor alone, this conclusion is limited by the paucity of data.

It is predicted that as climate change continues there will be an increase in hypoxia events because the stratification of water bodies increases the oxygen demand of organisms in warmer waters and accelerates the flow of nutrients into water bodies such as estuaries [129,141]. Ocean acidification and hypoxia are already evident in some coastal ecosystems around the world where hypoxic water is under saturated with respect to aragonite which has upwelled onto the continental shelf [31]. Laboratory and field experiments to test the synergistic impacts of ocean acidification and hypoxia are emerging areas of research.

It is also anticipated that other anthropogenic factors such as UV exposure [142] and pollutants [143] may act synergistically with ocean acidification, temperature, salinity and hypoxia to push organisms and ecosystems past their threshold limits. Despite this, very few studies have investigated the impact of multiple stressors in synergy with ocean acidification on molluscs.

Impacts of ocean acidification on predator-prey interactions and disease response have also been relatively unexplored despite evidence of the likelihood of weaker shells (Table 1) and reduced immune response in molluscs [58,144,145]. Over the next century the lack of consideration of the synergistic impacts of ocean acidification with other environmental stressors will lead to an underestimation of the effect of changing ocean chemistry on mollusc species.

(d) Exposure of adults to elevated CO_2_ during reproductive conditioning will result in positive carry-over effects passed from adults to their offspring and will increase the resilience of molluscs to ocean acidification:

The substantial influx in the number of studies which report the biological consequences of elevated CO_2_ on molluscs are limited by a lack of understanding about the potential of species to acclimate to the fluctuations of pH in their environment. Some molluscs may thrive in a high CO_2_ environment (such as adult *Sepia officialis* which increase calcification), some may be unaffected, while the large majority may face declines in abundance, habitat restructures and even extinction. In the few studies to investigate within population response to ocean acidification, differences in sensitivities have been reported. For example, Parker *et al.* [53] found that the shell growth of newly metamorphosed spat of a wild population of the Sydney rock oyster *S. glomerata* was reduced by 64% following a four day exposure to elevated CO_2_ (pH −0.4 from ambient), but was only decreased by 25% in a selectively bred population of the species (developed by aquaculture for faster growth and resistance to disease). Further, in a naturally occurring population of the blue mussel *M. edulis*, collected from Kiel Fjord in the Baltic Sea, where seawater *p*CO_2_ is elevated for large parts of the year due to upwelling of CO_2_, rich water, juveniles maintained normal somatic and shell growth rates following exposure for eight weeks to elevated *p*CO_2_ of 1,400 µatm [79]. In comparison studies on *M. edulis* from geographical locations, not naturally high in seawater, CO_2_ showed a range of negative effects including reduced calcification and shell growth in adults and larvae and reduced hatching success in embryos [45,60,100]. These studies suggest that some mollusc species may have undergone a pre-adaptation to elevated CO_2_ based on environmental phenotypic plasticity or evolutionary adaptation.

Nearly all of the studies on molluscs to date, have assessed the impacts of elevated CO_2_ on either the “benthic” (juveniles and adults) or “early-life” history stages. These studies allow an assessment of the sensitivity of each stage, but they do not determine whether carry-over effects (positive or negative) of ocean acidification can be passed from one stage to another. Indeed, the importance of carry-over effects in the evolutionary history of marine organisms has been documented for a number of mollusc species. In the oyster *C. virginica* the optimum salinity range for the development of embryos and larvae was largely influenced by the salinity that the adults were held in prior to spawning [146]. There is evidence from the only transgenerational ocean acidification study done on molluscs to date, that positive carry-over effects from adults to their offspring during exposure to elevated CO_2_ may give them the capacity to acclimate to the suboptimal conditions [38]. Parker *et al.* [38] found that *S. glomerata* exposed adults to elevated CO_2_ (pH −0.3 from ambient) during reproductive conditioning, facilitated acclamatory processes in larvae. Larvae produced from these parents were larger in size and developed at a faster rate under elevated CO_2_ when compared to larvae produced from parents conditioned under ambient CO_2_ conditions.

Such changes in phenotypic traits of offspring following exposure of adults to environmental stress may be linked to an adaptive maternal effect. Mothers may respond to environmental stress by increased maternal energy investment per offspring thereby increasing offspring size, a trait which is often considered to be beneficial during larval development and settlement [147,148,149]. Alternatively these offspring may have developed more efficient ion-regulating processes [37]. While Parker *et al.* [38] suggested a positive carry-over effect of elevated CO_2_, a recent study on a non-mollusc species (green sea urchin *Strongylocentrotus droebachiensis*) revealed negative carry-over effects from adults to their offspring [150].

The experimental design to test hypotheses on the carry-over effects of ocean acidification studies is often difficult, but is a key area for future research. The long generation times of many mollusc species make such studies difficult to do. If we are to predict possible carry-over effects in other mollusc species in a future high CO_2_ world, such studies are required.

## 4. Physiological Responses of Molluscs

Understanding the physiological responses of molluscs has been suggested as key in explaining whole organism responses. As the concentration of CO_2_ in seawater increases (hypercapnia) it readily diffuses across the epithelial surfaces of marine organisms where it equilibrates across the body spaces [74,151]. The increase in the concentration of CO_2_ in the body spaces, much like the reaction of CO_2_ dissolving into seawater, has an acidifying effect on acid-base balance. Once CO_2_ enters the organisms it reacts with internal body fluids and buffers in equilibrium with these fluids to form HCO_3_^−^ and H^+^ ions. The net effect of this reaction is an increase in the internal concentration of CO_2_, HCO_3_^−^ and H^+^ and a decrease in both intra- and extracellular pH [41,74]. If uncompensated, this disturbance in acid-base balance can have serious consequences for marine organisms including, changes in energy metabolism [40,43,51,137,152], reduced thermal tolerance and aerobic scope, reduced immune response [129], protein degradation [43], reduced somatic growth [40], and in extreme cases mortality [44,49,60,72,153].

During chronic exposure to elevated *p*CO_2_, as predicted in a future high-CO_2_ world, molluscs will rely on ion-exchange mechanisms to help maintain acid-base balance [74,154]. This involves the active transport of acid-base relevant ions such as H^+^ or HCO_3_^−^ across cell membranes using protein carriers such as Na^+^/K^+^-and H^+^-ATPases and dependent carriers such as Na^+^/H^+^-exchange, thereby facilitating the removal of H^+^ and accumulation of HCO_3_^−^ ions [41,74,154]. In nearly all mollusc species tested to date compensation of CO_2_-induced acid-base disturbances has involved an accumulation of HCO_3_^−^ ions [43,71,137,155]. Generally, however, benthic mollusc species with slow moving, hypometabolic modes of life (particularly bivalves and gastropods), have low ion-exchange capacities [41,156] and are therefore, limited in their ability to compensate for changes in acid-base status [41,74,75]. Incomplete compensation of acid-base status, namely pH_e_ has been identified as a major factor influencing physiological processes during hypercapnic stress [156]. This is particularly true for energy metabolism which is often depressed when pH_e_ is reduced [156]. A growing number of studies have investigated the effect of elevated *p*CO_2_ exposure on the energy metabolism of marine and estuarine molluscs [40,43,51,136,137,151,152,155,157,158,159,160,161,162] (Table 1). In many bivalve molluscs tested to date there has been as increase in standard metabolic rate (SMR) following exposure to moderately elevated *p*CO_2_ [38,40,137,152,157,158]. This was not the case for the mussels *M. galloprovincialis* [151,159], *M. chilensisi* (Hupe 1854) [160] nor for the pearl oyster *Pinctada fucata* (Gould 1850), green mussel *Perna viridis* (Linnaeus 1758) [158], clams *Ruditapes decussatus* [157] and *Acesta excavata* [161] and the periwinkle *Littorina littorina* [162]. Increases in SMR during exposure to elevated CO_2_ suggest that for some mollusc species there may be a higher cost of basal metabolism unless acclimation across life stages or evolutionary adaptation occur. A recent study by Beniash *et al.* [40] found increased SMR in the juvenile oysters, *Crassostrea virginica*, following a 20 week incubation at elevated *p*CO_2_ of 3,523.3 µatm (pH −0.7 from ambient). This increased SMR was also accompanied by higher mortality and slower shell and somatic growth. The authors suggested that slower somatic growth may be the result of a greater proportion of an organism’s energy being diverted from growth to basal metabolism [40]. Reductions in somatic growth were also reported in the oyster, *C. gigas* [137] during exposure to elevated *p*CO_2_ (pH −0.4 from ambient), but not in the mussel *Mytilus edulis* [51]. Increased SMR was also reported for the clam, *Laternula elliptica* (King and Broderip 1832) during exposure to elevated *p*CO_2_ [152]. Cumming *et al.* [144] exposed adult *L. elliptica* over 120 days to elevated *p*CO_2_ concentration of 735 µatm (pH −0.21 from ambient), a concentration much lower than Beniash *et al.* [40] and a pre-industrial *p*CO_2_ level of 187 µatm (pH +0.33 from ambient). The SMR of the clams increased in both the elevated *p*CO_2_ and low *p*CO_2_ (pre-industrial) compared to ambient treatments. Further in the elevated *p*CO_2_ treatment, gene expression of chitin synthase (CHS), a key enzyme in the formation of bivalve shells, increased indicating a stimulation of chitin synthesis. In the oyster, *C. gigas*, however, an increased SMR only occurred when the oysters were exposed to the combined effects of elevated *p*CO_2_ and elevated temperature [137]. When reared at elevated *p*CO_2_ of 1,480 µatm (pH −0.4), the SMR of adult *C. gigas* did not increase despite an incomplete compensation of pH_e_ (pH_e_ −0.49 from ambient). When reared at elevated *p*CO_2_ and elevated temperature (+5 °C), however, there was a significant increase in SMR. This indicates that despite a reduction in pH_e_ there was no elevated whole organism energy demand in the CO_2_-exposed animals when reared at their optimal temperature, but there was an elevated energy demand in CO_2_-exposed animals reared at an elevated temperature [138]. Incomplete pH compensation may suggest, however, that metabolic rate was depressed in some organs (e.g., muscle or hepatopancreas). This effect may be compensated for by the stimulation of energy demand in other tissues like gills.

Overall the suboptimal conditions induced by exposure of bivalve molluscs to ocean acidification scenarios may be associated with greater baseline energy costs in some tissues. During episodes of moderate stress these metabolic energy demands may be met through increased SMR (as seen in the bivalves above). In episodes of more extreme stress, however, the food supply available to an animal and/or the capacity of systemic functions may not be adequate to meet the needs for increased energy. When this occurs and compensation of energy homeostasis is not possible, an organism can enter a state of metabolic depression in an effort to conserve energy and prolong survival [137]. Such metabolic depression typifies the situation during air exposure at low tide [163]. Metabolic depression was measured in the mussel *M. galloprovincialis* following exposure to elevated *p*CO_2_ [43]. Michaelidis *et al.* [43] exposed juvenile and adult *M. galloprovincialis* for three months to CO_2_-induced acidification of seawater to pH 7.3 a level much lower than that predicted for the end of the century. The chronic exposure resulted in an uncompensated acidosis of pH_e_, despite an accumulation of HCO_3_^−^ [43] causing a reduction in SMR of the mussel and a net degradation of proteins [43]. Furthermore, the accumulation of HCO_3_^−^ to compensate for acidosis was partly from CaCO_3_ shell dissolution (as seen by an increase in Ca^2+^ in the hemolymph) [43] a phenomenon also reported in the mussel *M. edulis* [164]. Stimulated amino acid or protein degradation measured in *M. galloprovincialis* and other mollusc species *M. edulis*, [51] and *Patella vulgata* (Linnaeus 1758) [155] following ocean acidification stress may impact on processes such as somatic growth [40,51,137] and reproduction. Negative behavioural changes such as reduced clapping performance may also occur as shown in *Pecten maximus* (Linnaeus 1758) [165]. Under short-term (acute) exposure to environmental hypercapnia, reduced SMR can act as a survival mechanism, allowing marine organisms to tolerate unfavourable conditions such as low tide. After prolonged (chronic) exposure, however, reduced SMR in *M. galloprovincialis* may become lethal. Reductions in survival have been also documented in a number of mollusc species following prolonged exposure to elevated *p*CO_2_ (gastropod *Strombus lubuanus* (Linnaeus 1758) [44]; bivalves including mussel *M. edulis* [60]; clams *Mercenaria mercenaria* [141]; *Ruditapes decussates* (Linnaeus 1758) [69]; oyster *C. virginica* [40]; pteropod *Limacina helicina* (Phipps 1774) [49]).

In the only gastropod species studied to date, there was no change in SMR in the limpet, *P. vulgata* following exposure to CO_2_-acidified seawater and a pH of 7.6 (pH −0.6 from ambient) [155]. While the limpet sufficiently compensated for extracellular acidosis by the accumulation of extracellular [HCO_3_^−^] and an increase in extracellular [Ca^2+^], the source of HCO_3_^−^ ions involved the dissolution of the shell [147] in a process likely similar to that for *M. galloprovincialis* [43].

Finally, studies on the physiology of the cephalopod mollusc *S. officinialis* show that this species is quite robust to ocean acidification stress. In contrast to bivalves and the gastropod, exposure of the cuttlefish to elevated *p*CO_2_ of 4,000 and 6,000 ppm had no effect on SMR or growth [67] with pH_e_ remaining somewhat lower than the controls and being compensated through the active accumulation of HCO_3_^−^ ions to a greater extent than that seen in bivalves [71]. Further, the accumulation of HCO_3_^−^ ions does not seem to involve CaCO_3_ dissolution, unlike in the gastropod and bivalve species. Not all cephalopod molluscs, however, show a tolerance to ocean acidification. In the only other cephalopod species studied to date the jumbo squid *D. gigas* exhibited a depressed metabolic state and a reduction in activity levels when exposed to seawater acidified to 7.8 (pH −0.3 from ambient) [136]. 

Overall, the complex nature of the physiological responses of molluscs to ocean acidification requires further investigation. The changes in SMR documented for many mollusc species may reflect an impact on energy turnover and allocation to fitness sustaining processes [156], including growth and repair [40,43,51,53,72], immune response [58,143,145] and reproduction (no studies to date). Through an understanding of the underlying mechanisms involved in species responses we will get a greater understanding of “how” and “why” there is such variability in responses both within and between closely related species.

## 5. Food Availability and Energy “Trade-offs”

The capacity for marine and estuarine molluscs to acclimate to the continuing changes in ocean chemistry over the next century may be largely reliant on their ability to meet energetic needs. It has been predicted that there will be a higher energetic cost of routine metabolism for many mollusc species as our oceans continue to acidify. Already, as described in the previous section, a limited number of studies have found that in nearly all molluscs tested to date, exposure to moderately elevated levels of CO_2_, caused increased standard metabolic rate (SMR) [38,40,136,152,164], suggesting that for some mollusc species there may be a higher energetic cost of routine metabolism, as molluscs attempt to maintain acid-base balance in a high-CO_2_ world.

Molluscs may be able to compensate for this higher energetic cost of routine metabolism during exposure to ocean acidification when food availability is high because of increased energy intake and assimilation. When food availability is low, however, such compensation may be incomplete or impossible because the synergistic impact of ocean acidification and low food availability may cause a greater disturbance in acid-base balance and mediate unfavourable “trade-offs” in the energy budget. If this occurs then there will be negative consequences on critical fitness sustaining processes such as shell and somatic growth, immune response and reproduction as molluscs attempt to maintain ion and acid-base balance and prolong survival. In the only study so far to consider the impact of ocean acidification with altered food availability, Melzner *et al.* [166] found greater internal shell dissolution of the mussel, *M. edulis* during exposure to elevated CO_2_ when food availability was low compared to when food availability was high. Further, Thomsen *et al.* [65] found that when food concentration is high, growth and survival of newly settled *M. edulis* is not affected. This highlights the importance of food availability and the energy budget of molluscs under high CO_2_-stress. The responses of mollusc species to ocean acidification may depend on the level of primary productivity and food availability which will differ across geographic regions and/or seasons. Primary productivity is already believed to constrain the amount of fish and invertebrates available to expanding fisheries [167], and it is likely to be even more significant in a climate changed ocean. Alterations in the partitioning of the energy budget of molluscs, especially during energetically costly times such as reproduction, have been virtually unexplored. These studies are necessary in order to fully predict which energy trade-offs will occur within mollusc species.

## 6. Conclusions and Future Research

**Figure 2:**
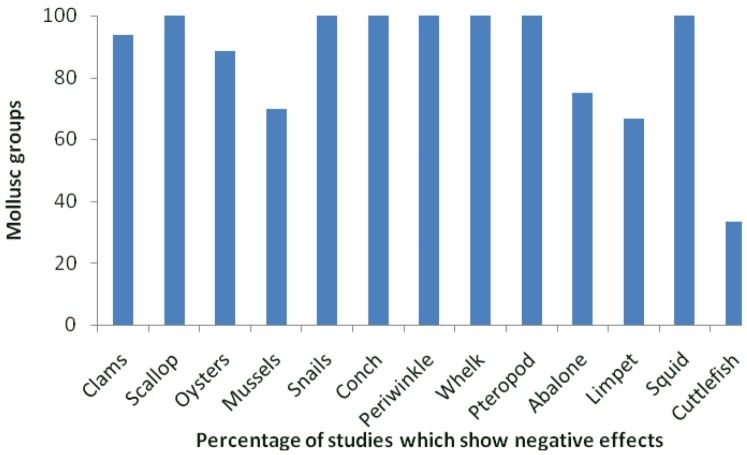
Percentage of studies which show negative effects of ocean acidification on one or more processes for each mollusc group.

Over coming decades, it is likely that ocean acidification will pose serious consequences for many marine and estuarine molluscs (Figure 2). A review of the evidence to date suggests that while more evidence is needed before conclusions can be made it is likely that fertilisation, embryonic, larval development, settlement and juvenile and adult calcification will be highly vulnerable to elevations in CO_2_. The most sensitive life-history stage seems to be larvae, with nearly all studies reporting negative impacts on this critical stage of development. Calcification and SMR of bivalves and gastropods has found to be most impacted, with cephalopod molluscs more tolerant of ocean acidification with no impact on adult calcification or SMR [67,71,115,136], perhaps due to greater ion-exchange capacities in cephalopods, which are not well developed in bivalves and gastropods.

Even sub-lethal effects of elevated *p*CO_2_ can severely alter the composition and fitness of marine populations. Common impacts of ocean acidification on the early developmental stages of most mollusc species studied are a reduction in the rate of larval development and larval size (Table 3), both of which potentially have consequences for the survival of mollusc species. Prolonging the length of larval life increases the chance of predation in the water column, particularly in the absence of properly calcified shells and skeletons and reduces the time available to settle. Furthermore, reduced larval size can reduce the feeding efficiency of larvae [76] (although there is no evidence for this in the context of ocean acidification to date), as previous studies have suggested that smaller larvae are more susceptible to starvation than larger larvae as they encounter comparatively less food [76,168,169,170].

In many studies the responses of molluscs are measured in experiments where the duration of exposure is acute (sudden drop in pH of 0.4 units) which does not mimic well the non-acute, longer time frame expected for oceans to acidify. Results from acute experiments make it difficult to extrapolate to longer term impacts. Extrapolation is also difficult because results from the laboratory, where the majority of work has been done are not necessarily replicable in the field [14]. There have been only two studies to date which directly considered the impact of ocean acidification on the settlement of bivalves and gastropods in the field. Cigliano *et al.* [126] placed artificial collectors along a pH gradient, ranging from 7.08–8.15, created by CO_2_ vents off the coast of Ischia in the Tyrrhenian Sea, Italy. After one month, they found a significant reduction in the recruitment of a range of bivalve and gastropod species as the seawater pH decreased from normal (8.09–8.15) to low (7.08–7.79), suggesting that settlement of benthic molluscs may be highly impacted as our oceans continue to acidify. Studies on juvenile abundance and adult shell strength done at the same location further support these results. Juveniles of the gastropod snails *Osilinus turbinate* (Born 1778) and *Patella caerulea* (Linnaeus 1758) were absent from sites with very low pH (pH ≤ 7.4) but were present at the site with normal pH (pH 8.09–8.15) [46]. Further, the shell strength of adult snails *Hexaplex trunculus* (Linnaeus 1758) and *Cerithium vulgatum* (Bruguière 1774) was reduced in acidic seawater [46]. 

As many of our conclusions to date are based on the results of single species, single factor studies, our current understanding of the biological consequences of an acidifying ocean are dominated by large uncertainties. In order to fully understand the consequences of ocean acidification at the population and ecosystem level, multi-generational and multi-stressor experiments on species from different geographic locations are needed to assess the adaptive capacity of mollusc species and the potential winners and losers in an acidifying ocean over the next century. Future research needs to move away from single-species responses on one stage in the lifecycle and consider the synergistic effects of multiple stressors (*i.e.*, temperature, hypoxia, food concentration) on different life-history stages and the potential for species to acclimate or adapt. The measurement of the underlying mechanisms responsible for adaptation or acclimation is essential if we are to fill the gaps in our understanding and maintain the ecological and economic services provided by this diverse phylum.

Finally, recent studies are addressing effects of ocean acidification on species at local and regional scales. This is of enormous worth because while ocean acidification is a global problem there will be local regions and/or habitats which are likely to be affected more than others. This may be through exposure to greater, faster reductions in pH (in areas of upwelling, estuaries or high latitude regions where atmospheric CO_2_ absorption is expected to be greater) or predisposition to other environmental stressors (temperature, salinity, hypoxia). Areas of future research described in this review will make modelling of the biological impacts of ocean acidification more possible and allow for the development of policy on the socio-economic impact on fisheries and aquaculture to sustain the global populations of molluscs.
